# Label-free volumetric imaging of porcine kidney tissue over extended areas using dynamic MHz-OCT

**DOI:** 10.1038/s41598-025-15032-6

**Published:** 2025-09-12

**Authors:** Sazgar Burhan, Madita Göb, Mario Pieper, Tjalfe Laedtke, Thorge Grahl, Michael Münter, Hinnerk Schulz-Hildebrandt, Gereon Hüttmann, Peter König, Robert Huber

**Affiliations:** 1 Institute of Biomedical Optics, Universität zu Lübeck, Peter-Monnik-Weg 4, 23562 Lübeck, Germany; 2 Medizinisches Laserzentrum Lübeck GmbH, Peter-Monnik-Weg 4, 23562 Lübeck, Germany; 3 Institute of Anatomy, Universität zu Lübeck, Ratzeburger Allee 160, 23562 Lübeck, Germany; 4 Airway Research Center North (ARCN), German Center for Lung Research (DZL), Lübeck, Germany; 5 Mass General Brigham, Wellman Center for Photomedicine, Harvard Medical School, 55 Fruit Street, Boston, MA 02114 USA

**Keywords:** Imaging and sensing, Kidney

## Abstract

Dynamic optical coherence tomography (dOCT) enables high-resolution, label-free 3D tissue imaging with functional contrast, offering a potential advancement in kidney transplant viability assessment. Currently, no widely accepted non-invasive method provides an objective evaluation of transplant viability, as conventional imaging lacks the resolution to assess microvascular integrity and cellular dynamics. However, the clinical use of available dOCT systems is limited by prolonged imaging times that hinder clear visualization of tissue dynamics. To address these challenges, this paper presents a swept-source 3.2 MHz-OCT system for dynamic, high-speed, extended-area imaging. The system integrates an optimized inter-volume scan protocol and a micrometer-precision linear robot, enabling extended‑scale tissue imaging over an area of about 2.6 × 2.6 mm^2^. Using freshly excised porcine kidney tissue, we demonstrate recent advancements and challenges in MHz-dOCT for kidney imaging. Identified anatomical features are thoroughly examined, compared across resolutions, and validated through histology to assess the system’s clinical potential.

**Supplementary Information:**

The online version contains supplementary material available at 10.1038/s41598-025-15032-6.

## Introduction

The growing disparity between the demand for kidney transplants and the availability of donor organs has become a critical challenge in the management of patients with end-stage renal disease^1^. Due to prolonged waiting times, surgeons often need to consider using organs from expanded criteria donors (ECD), which carry a higher risk of suboptimal function or graft failure^2–4^. The success of transplantation, however, is critically dependent on the quality and viability of the donor kidney.

Current imaging modalities, such as Doppler ultrasound and computed tomography angiography, can assess kidney viability only before organ retrieval. Once the kidney is explanted, no reliable imaging method exists to predict its post-transplant function. As a result, clinical practice often relies on subjective visual inspection, evaluating general appearance, texture, signs of damage, and visible abnormalities^1^. Yet, this approach is inherently subjective, lacks consistency, and fails to provide quantitative insights into the organ’s structural and functional condition. Pre-transplant biopsies offer a more objective alternative by assessing microstructural integrity, particularly the percentage of glomerulosclerosis and tubular necrosis, both associated with poor graft outcomes^5^. However, histological analysis is time-consuming, potentially affecting tissue viability, and is limited to specific sample sizes, increasing the risk of sampling errors^5^. Given these challenges, there is an urgent need for a real-time imaging technique capable of assessing kidney viability immediately after retrieval. In an ideal scenario, this method should provide extended-area imaging with histology-like resolution, enabling a non‑invasive evaluation of the kidney’s microstructural integrity. Such an approach would provide immediate, quantitative insights into the organ’s structure and suitability for transplantation without requiring invasive biopsies or risking the loss of the entire kidney.

One promising technique that could fulfill this need is optical coherence tomography (OCT), a widely used imaging technique in medical diagnostics. OCT offers non-invasive, high-resolution volumetric images of biological tissue structures^6^. Based on the principle of low-coherence interferometry, the acquisition of detailed structural information at a micrometer scale is possible^7^. Several studies have demonstrated the feasibility of OCT for kidney imaging^8–11^, including measuring structural tubular parameters and quantifying microcirculation in both pre- and post-transplant assessments.

Building on OCT’s success in structural imaging, label-free functional OCT extensions have further enhanced its potential by capturing not only static features but also dynamic tissue changes. Recently developed techniques, such as logarithmic intensity variance contrast^12^ and dynamic OCT (dOCT)^13–16^, exploit metabolically driven scattering dynamics to detect intracellular motion. These methods reveal changes in optical backscattering properties by repeatedly acquiring images over several seconds and analyzing temporal signal fluctuations at the voxel level. When specific color values are assigned to temporal fluctuations in the OCT images, it provides a visually intuitive, histology‑like view of tissue architecture and activity. Successful demonstrations of dOCT have been performed with both full-field^14,17^ and spectral-domain^15,18^ OCT systems. Fast scanning dOCT has been applied to in vitro organ cultures^14^, ex vivo studies of mouse trachea^19,20^, and human otologic pathologies^21^, demonstrating its potential to enhance the analysis of physiological and pathological processes. However, current full‑field and spectral-domain OCT systems face constraints mainly due to the camera’s sensor readout speed, restricting A-scan rates to the hundreds of kilohertz range^22^. This results in small imaging areas, prolonged imaging times, and significant motion artifacts, which are particularly problematic for dOCT, where motion artifacts can introduce false dynamic signals. For real-time applications, an OCT system with A‑scan rates in the MHz range is needed to provide live feedback and extended-area scans. Furthermore, high-speed imaging enables rigid volume acquisitions, simplifying subsequent numerical sample motion corrections^23^.

With the introduction of Fourier Domain Mode Locking (FDML) lasers in 2006^24^, MHz-OCT has emerged as a promising solution to overcome speed limitations^25–29^. These systems can achieve A-scan rates of up to 5.2 MHz with single-spot and 20 MHz with parallel four-spot imaging^28^, significantly reducing motion artifacts, enabling real‑time imaging at video rates^26,27^. Despite these advantages, the application of MHz‑OCT for dynamic OCT imaging remains largely unexplored, as several technical challenges must still be addressed. One of the primary limitations of current MHz-OCT systems is their relatively low axial resolution of approximately 16 µm, which may hinder the detailed visualization of fine cellular structures. Additionally, the high scanning speed introduces revisitation errors, leading to insufficient phase stability between repeated B-scans that can compromise the system’s ability to capture dynamic processes accurately. Another major challenge is the limited field of view (FOV) associated with high lateral resolution. Objectives with a high numerical aperture (NA) are required for capturing fine details, but their use inherently restricts the imaging area. Instead of switching to lower-resolution scan lenses, which would compromise image quality, an alternative approach is necessary to achieve extended-area imaging. This requires precise positioning of the scan head across the sample to enable a comprehensive assessment of the tissue. By acquiring multiple adjacent volumes and stitching them together, the effective scanning area can be expanded without sacrificing spatial resolution.

This paper investigates these challenges and presents the implementation of a swept-source MHz-dOCT system with an optimized inter-volume scan protocol for extended-area dynamic imaging. The system integrates a home-built, motorized, three-axis linear robot to enable extended area scanning. Using freshly excised porcine kidney tissue, we demonstrate the system’s capability to capture dynamic cellular contrast and explore its potential for pre-transplant kidney assessment. A detailed analysis of different optical resolutions for imaging renal tissue structures is conducted to evaluate the system’s imaging capabilities. Additionally, the anatomical features identified through MHz-dOCT are examined in comparison with histological validation to assess the accuracy and clinical relevance of the technique. By systematically addressing the technical limitations associated with MHz-OCT, this study aims to advance the method as a real-time, high-resolution imaging tool for kidney viability assessment. The findings contribute to the development of an alternative approach to traditional histology, offering the potential to improve the evaluation of donor organs and enhance transplant outcomes.

## Results

### Extended field of view dOCT imaging by motorized scan head positioning

**Fig. 1:**
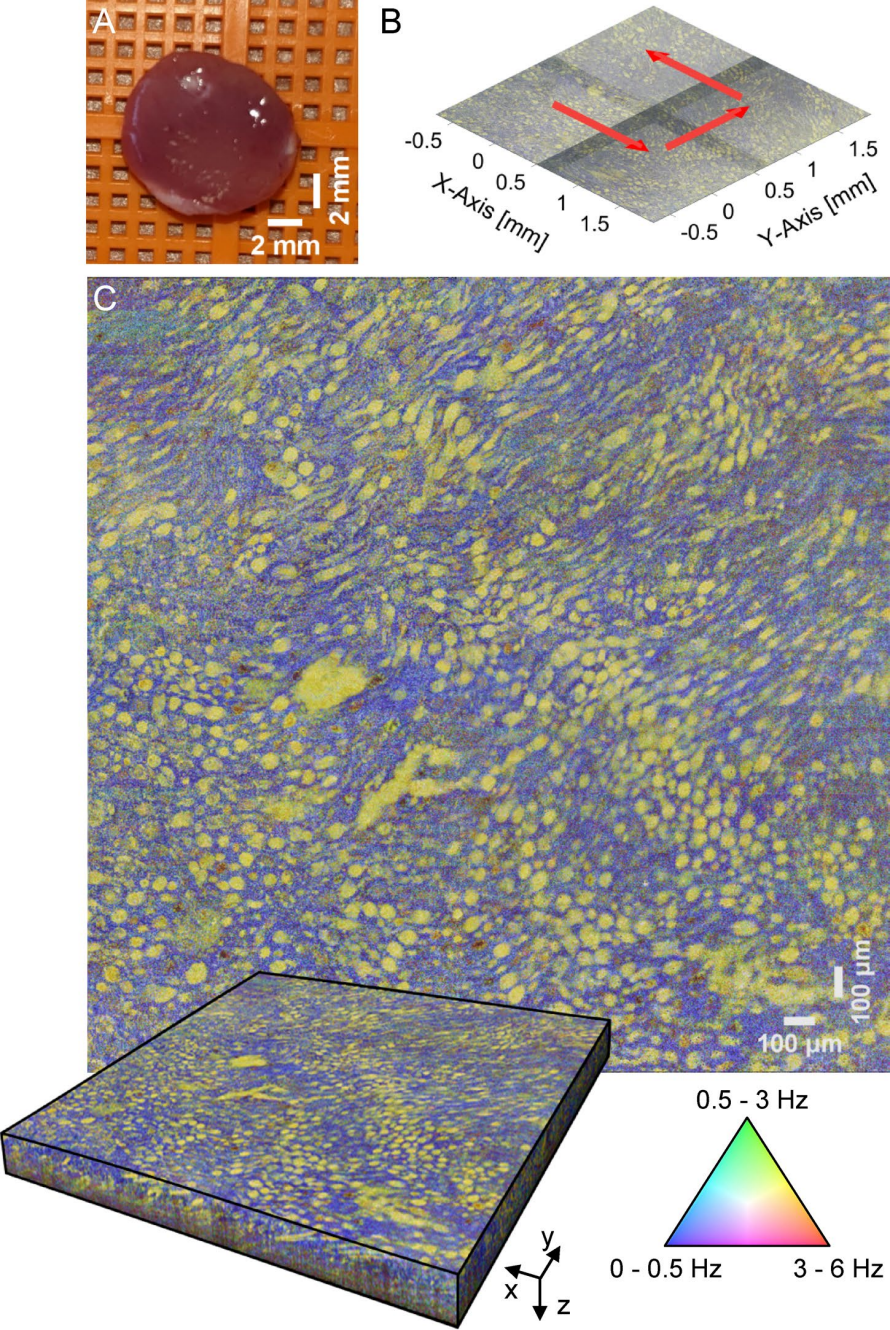
Extended field of view dOCT imaging using a precise three-axis linear robot. A: Image of the sample extracted from the inner medullary region. B: Movement path of the linear robot. C: Stitched 2.6 × 2.6 mm^2^ cross-sectional *en face* dOCT image at a 40 µm depth, along with the volumetric image of the ex vivo porcine kidney, clearly visualizing the renal collecting duct system.

Figure 1A presents a porcine kidney sample extracted from the renal pyramids within the inner medullary region. The renal pyramids are cone-shaped structures essential for kidney function as they facilitate urine formation and collection. Each pyramid contains several cellular and microstructural components, including collecting ducts and thin loops of Henle. The loop of Henle creates a concentration gradient through water and ion reabsorption, allowing the collecting ducts to concentrate urine before transporting it to the renal papilla at each pyramid’s apex and onward into the renal pelvis. This process plays a pivotal role in fluid and electrolyte balance.

A home-built motorized three‑axis linear robot was used to achieve precise, micrometer-level positioning across multiple locations. The scan head was sequentially positioned at four distinct points, as indicated in Fig. 1B, where red arrows illustrate the order and direction of movement. Using a 10 × microscope objective, each scan captured a lateral FOV of 1.4 × 1.4 mm^2^, with an approximately 15% overlap between adjacent volumes to ensure seamless stitching and uninterrupted spatial coverage. Figure 1C displays the dOCT result with the corresponding color scale. A stitched cross-sectional *en face* image at 40 µm depth is shown alongside a rendering of the stitched three-dimensional scan. The resulting composite three-dimensional dataset has a total FOV of approximately 2.6 × 2.6 mm^2^, densely sampled with 4096 × 4096 × 600 voxels.

The dOCT results successfully reveal detailed microscopic features of the kidney. Fast signal variations (3–6 Hz) are shown in red, medium frequencies (0.5–3 Hz) in green, and blue colors indicate slow signal variations (0–0.5 Hz). The dynamic contrast observed in the ex vivo kidney tissue can be attributed to intrinsic cellular motion, which persists for several hours post‑excision. This residual movement may arise from several mechanisms, including thermally induced diffusion and Brownian motion, both of which contribute to subtle yet significant fluctuations in the optical scattering properties of the tissue. The dOCT images reveal well-defined morphological details and structural organization within the renal medulla. Distinct yellow-green structures are delineated against a blueish background. Their rounded and elongated shapes are consistent with the expected cross‑sectional appearance of collecting ducts and Henle’s loops. Scrolling through successive cross-sections reveals the spatial organization of the renal pyramids, including the progression of ducts and loops, as shown in the three-dimensional animation provided in the supplementary material (Vid. S1). However, direct differentiation between these structures within the dOCT images remains challenging, as they only differ in diameter and not in contrast signal.

It is important to note that the subtle discontinuities observed in the stitched dynamic volumes are not caused by mechanical inaccuracies of the robot. Rather, these discontinuities likely represent minor stitching artifacts resulting from slight misalignments during the registration of adjacent subvolumes and do not indicate any instability of the scanning system, which has demonstrated stable performance and is not the source of the observed effects based on our evaluations.

### Comparative evaluation of imaging performance at different resolution levels

**Fig. 2:**
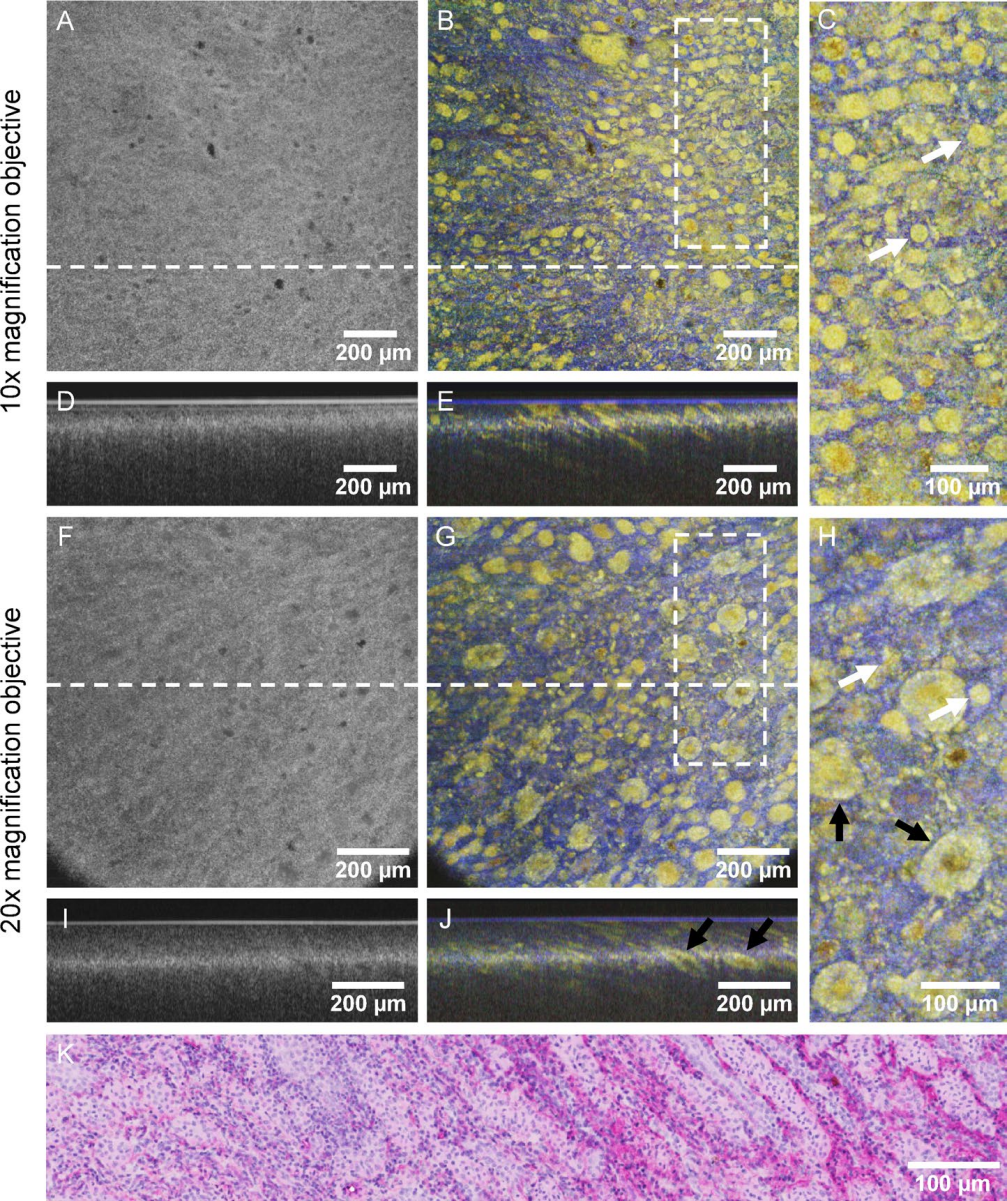
Comparative evaluation of different microscope objectives for imaging the inner renal medulla. A comparative analysis was performed with ×10 (**A**–**E**) and ×20 (**F**–**J**) magnification objectives. The OCT intensity images are presented in (**A**) and (**F**), alongside the corresponding dOCT results displayed in (**B**) and (**G**). Magnified views of the regions highlighted in white in (**B**) and (**G**) are shown in (**C**) and (**H**). In addition, (**D**) and (**I**) present cross-sectional B-scan views of the intensity results, while (**E**) and (**J**) show the dOCT results at the positions indicated by the white dashed lines in (**B**) and (**G**). White arrows illustrate selected Henle’s loops, and black arrows mark collecting ducts. The corresponding H&E-stained histological section is presented in panel (**K**), showing densely packed cells with dark-stained nuclei and a fibrous, eosinophilic extracellular matrix. This reflects the organization of the collecting ducts and Henle’s loops, with some areas appearing more structured, while others exhibit a more dispersed cellular arrangement.

To determine the optimal lateral resolution required for precise visualization of cellular structures, a comparative analysis was performed using 10 × and 20 × magnification objectives, with NAs of 0.26 and 0.4, respectively. The results obtained with the 10 × objective are presented in Fig. 2A–E, while Fig. 2F–J shows the findings with the 20 × objective. Figures 2B, G display cross-sectional *en face* dOCT images extracted at 59 µm sample depth using the 10 × objective and 143 µm depth using the 20 × objective. For comparison, Fig. 2A, F present the corresponding standard OCT intensity *en face* images, which were processed similarly to the dOCT datasets but without functional calculations. The white dashed lines in the *en face* images mark the corresponding B-scan positions, illustrated in Fig. 2D, I for the intensity images and in Fig. 2E, J for the dOCT images. The white rectangles denote two areas depicted enlarged in Fig. 2C, H. Figure 2K displays a histological section of the analyzed tissue. All OCT measurements and histological sectioning were performed on the same kidney excision from the inner medullary region, as shown in Fig. 1A. It is important to note that while the OCT measurements and histological analysis were conducted on the same sample and targeted approximately the same central area, exact colocalization (including between 10× and 20× magnification) was not achieved due to inherent differences in tissue processing, sectioning, and imaging techniques, as well as the mechanical reconfiguration of the OCT scan unit required for the two resolution settings.

The 10 × objective offers a broader FOV of 1.4 × 1.4 mm^2^, enabling imaging of larger tissue areas. Additionally, its Rayleigh length is twice that of the 20 × objective, resulting in an enhanced depth of view in the OCT B-scans. The comparison of Fig. 2E, J shows that B-scans acquired with the 10 × objective reveal more depth detail than the 20 × B-scans, where characteristic features are only distinguishable at the focal depth. However, this improvement in depth comes at the expense of reduced lateral resolution. In contrast, the 20 × objective with a more restricted FOV of 1 × 1 mm^2^ and a narrow focal range in depth delivers enhanced resolution, essential for precisely visualizing finer cellular structures in *en face* view. This trade-off between FOV and resolution is further impacted by vignetting artifacts with the 20× magnification, resulting from suboptimal light guidance into the objective, causing shadowed areas in the lower left and right corners.

Cellular structures unrecognizable in the static OCT intensity images become distinctly visible in the dOCT images. This difference is particularly evident in the B-scans, where the intensity images reveal minimal or no identifiable cellular features. In contrast, the dOCT B‑scans prominently display these features, enabling detailed and precise evaluation. Similarly, while the *en face* intensity images may hint at the presence of cellular structures, they lack the clarity and detail of the *en face* dOCT images, which offer a much more comprehensive and accurate visualization of these cellular structures.

With both the 10 × and 20 × objectives, round and elongated shapes are observable down to diameters of just a few micrometers. Comparable structures are also evident in the corresponding histological section, as shown in Fig. 2K. In the magnified *en face* views in Fig. 2C, H, selected features are highlighted by white arrows. These dimensions align with the diameters of Henle’s loops, which typically range from 20 to 50 µm^30^. Furthermore, even smaller structures, potentially additional cellular components, are visible. At 20× magnification, the internal structure of larger collecting ducts (marked with black arrows in Fig. 2H, J) is distinguishable, with a light-green surrounding area and orange internal regions. The light‑green color corresponds to the tubular structure of the ducts, which are composed of compact cells that show medium signal frequencies. In contrast, fluid movements within the ducts correspond to higher-frequency signals, represented by orange-colored features. In the B-scan views, the characteristic elongated shape of the ducts is apparent. Particularly outside the focal range, dOCT exhibits superior contrast, enabling the identification of structures that are hardly distinguishable with standard intensity OCT.

Visualizations of the complete three-dimensional datasets are provided in the supplementary material for improved clarity. Animations in Vid. S2 and Vid. S3 emphasizes the differences between standard intensity OCT and dynamic OCT by juxtaposing detailed views of the datasets.

### Dynamic microscopic visualization of anatomical features in the renal cortex

**Fig. 3:**
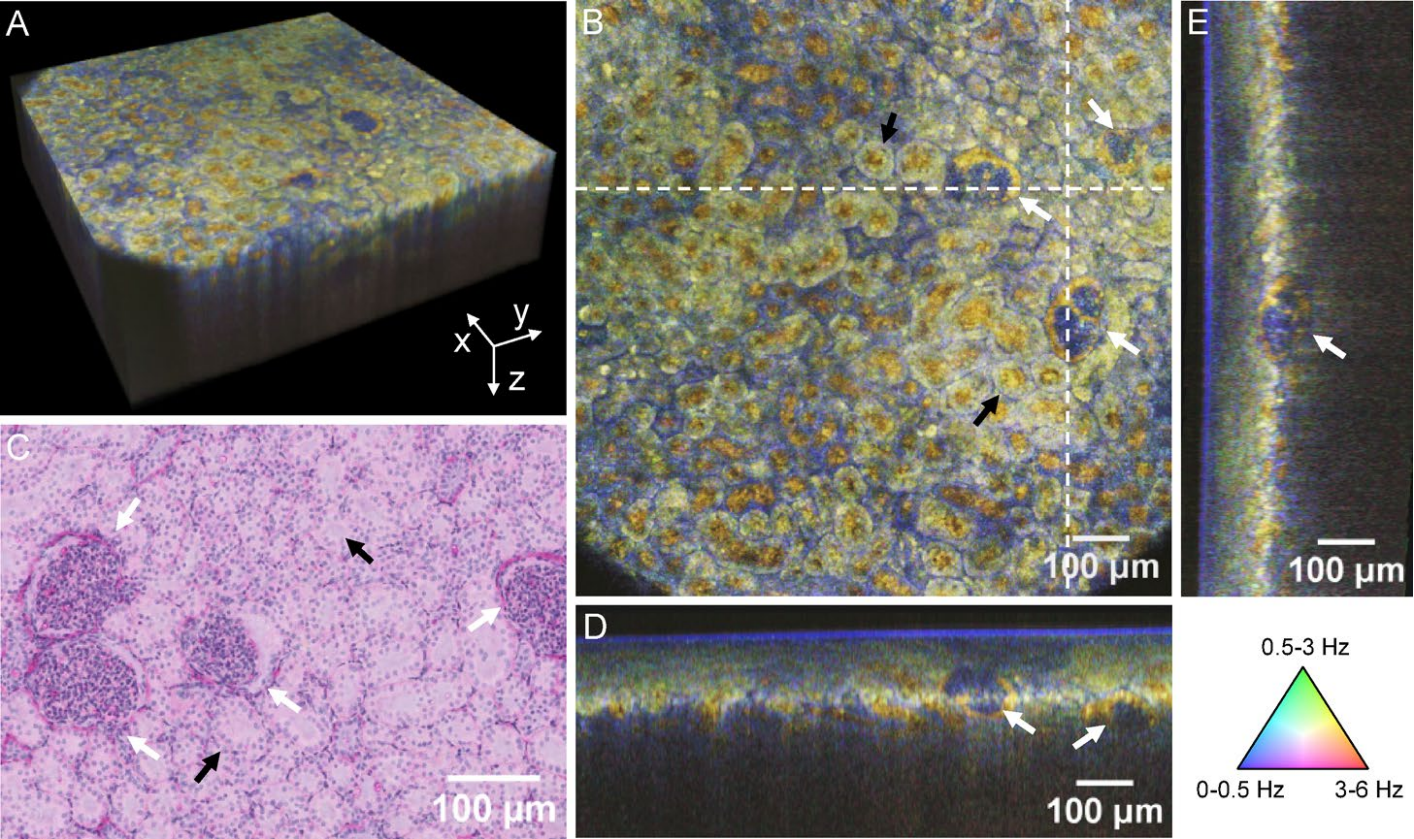
Dynamic OCT results from the outer renal cortex and histological comparison. (**A**) The three-dimensional dOCT dataset was acquired using a 20× magnification objective, exhibiting vignetting artifacts due to suboptimal light guidance in the objective. (**B**) Cross-sectional *en face* view extracted at 113 µm depth, with white dotted lines marking the positions of the orthogonal cross-sectional views shown in (**D**) and (**E**). (**C**) Corresponding H&E-stained histological section that exhibits densely packed cells with dark-stained nuclei predominantly within the glomeruli and tubular walls, with an eosinophilic extracellular matrix surrounding the structures. White arrows mark renal corpuscles, and black arrows indicate selected convoluted tubules. The discrepancy in the scale bars is attributable to sample shrinkage during histological processing.

In addition to the inner renal medulla imaging, measurements were also performed on the outer renal cortex to analyze the ability to visualize superficial structural details. A histologic comparison was performed to verify and correlate the dOCT findings. Figure 3A illustrates the resulting dOCT volume acquired with 20× magnification, while Fig. 3B displays a dOCT cross-sectional *en face* scan extracted at 113 µm depth. White dotted lines mark the position of the orthogonal cross-sectional B-scan views, shown in Fig. 3D, E. A corresponding histological section of the analyzed tissue is provided in Fig. 3C.

Various anatomical structures are visible in the dOCT images, with renal corpuscles being particularly prominent, indicated by white arrows in the figure. The dOCT imaging results clearly differentiate between the glomerulus and its surrounding Bowman’s capsule. The glomerulus, a tuft of capillaries where primary kidney filtration occurs, appears blueish in the images and is characterized by a low-frequency signal, likely due to the densely packed, mostly static cells within these structures. In contrast, the space between the glomerulus and Bowman’s capsule, known as Bowman’s space, appears yellowish, suggesting a different scattering property attributable to its liquid-filled, cell-poor composition. These contrasting optical properties enable the precise identification of individual components.

In addition to the corpuscles, yellowish structures in the dOCT volume represent convoluted tubules, which actively reabsorb essential solutes and water. Selected tubules are marked with black arrows in the figure. Although this ex vivo sample had been removed from the organism for several hours, the residual molecular movement remains detectable, producing a yellowish, higher-frequency contrast in the center of the tubules. In contrast, the surrounding areas appear less intense and green, corresponding to lower-frequency motion. Additional details of the three-dimensional dOCT dataset are presented in Vid. S4 within the supplementary material. Sequential virtual slicing in three orthogonal directions, combined with zoomed-in views, demonstrates the capability of the presented imaging technology to differentiate cellular structures through dynamic contrast in 3D. The results indicate that although the corpuscles are identifiable in the B-scans, precise localization and detailed morphological analysis require three-dimensional reconstructions. In addition, the morphology of the renal tubules is only discernible from the *en face* view.

Histology confirms the presence of convoluted tubules and corpuscles, whose observed sizes align with the reported range between 100 and 250 µm^31^. However, it is important to note that the scale bars in the dOCT images and histological sections are not directly comparable. This discrepancy is due to histological processing, which was experimentally determined to introduce an approximate shrinkage factor of 1.5 to 2. Adjusting the scale bar to account for this variation is challenging, as the shrinkage is not uniformly distributed across the sample.

## Discussion

Considering the urgent need for an extended-area, microscopic, non-invasive imaging technique to assess pre-transplant kidneys in real-time, MHz-dOCT presents a promising solution. Imaging experiments on freshly excised porcine kidneys demonstrate the ability to identify various renal structures using dynamic contrast. In particular, Fig. 3 and Vid. S4 emphasize its potential for pre-transplant imaging, as the experiments replicate non-invasive imaging conditions while maintaining the integrity of the organ. Compared to previously reported slow, single-slice imaging techniques, the results suggest that precise localization and detailed morphological analysis necessitate three-dimensional reconstructions.

This paper presents the first application of a swept-source MHz-dOCT system for dynamic tissue measurements, featuring an optimized scan protocol for inter-volumetrically based dynamic contrast. The findings confirm that the MHz-dOCT system can visualize microscopic dynamic processes in biological tissue. Cellular structures within the renal medulla, such as collecting ducts and complex features of the renal cortex, including the components of renal corpuscles, were successfully revealed. Therefore, the MHz-OCT system’s axial resolution of approximately 16 µm for renal tissue appears adequate.

The lateral resolutions of 3.48 µm (10 × objective) and 2.76 µm (20 × objective) are sufficient to capture cellular-level structures. The 10 × objective increased both the FOV and depth of focus. In contrast, the 20 × objective displayed fine structures within the focal range more effectively. The enhanced lateral resolution was particularly advantageous for detailed imaging of the corpuscles. Moreover, dynamic volumetric imaging provides a comprehensive and spatially accurate view of anatomical structures, including the renal tubules, which are indistinguishable in some cross-sectional views. This capability is essential for precisely assessing kidney morphology, functionality, and overall viability. However, due to the optical penetration depth of only 1–2 mm, the proposed imaging technique is restricted to superficial imaging of the renal cortex. In vivo assessment of deeper medullary regions would require the development of needle probes^32^.

Compared to previous studies exploring OCT for kidney transplant imaging^8–11^, the introduction of dynamic contrast in this work significantly enhances OCT’s ability to visualize characteristic anatomical structures that are not readily resolved with standard OCT. Notably, the clear depiction of renal corpuscles, with well-defined glomeruli, Bowman’s space, and capsule, may provide valuable insights into the extent of glomerulosclerosis. Importantly, the dynamic contrast in MHz-dOCT offers more than enhanced color coding. It introduces endogenous motion-based contrast that provides access to functional tissue properties such as perfusion, intracellular motion, and fluid dynamics within the renal tubules. Similar to Wierwille et al., who utilized Doppler OCT to quantify glomerular microcirculation in vivo to evaluate the kidney condition^33^, the presented method could also assess perfusion dynamics in pre-transplant kidney tissue. Furthermore, the ex vivo findings of this work indicate that MHz-dOCT may detect alterations in the glomeruli after organ retrieval. In addition, the presented technique could also support automated quantification methods, as demonstrated in the segmentation algorithms developed by Li et al. and Konkel et al. for measuring kidney microstructures^9,11^. Especially, parameters like the tubular diameter provide potential diagnostic information to identify tubular necrosis and could thus serve as novel imaging biomarkers for assessing the kidney’s viability. Unlike conventional imaging, MHz-dOCT offers not only a comprehensive 3D overview of the tubular structures but also enables visualization of subtle microstructural dynamics that were previously inaccessible, thereby representing a meaningful technical advancement for renal tissue assessment.

However, to establish a direct correlation between dOCT signal features and kidney function, systematic longitudinal imaging at multiple time points after organ retrieval, combined with region-specific histological analysis using established markers, would be necessary. Such studies require dedicated pathological models and precise co-registration protocols and are thus beyond the scope of this technical demonstration. Nevertheless, the current work lays a critical foundation for future investigations aiming to link dynamic optical signals to functional or diagnostic outcomes.

The MHz-dOCT system acquired 40 sub-volumes comprising 50 repeated volumes within a scanning time of less than three minutes. The applied scanning protocol allowed dynamic visualization of changes at frequencies up to 6 Hz. The volume rate and frequency bins were thoroughly chosen based on previous experiments that applied dOCT processing on repeated line scans. As the sample exhibited minimal motion at high frequencies, a volume rate of 12 Hz was sufficient to detect major dynamic structures in renal tissue. However, frequency changes in the red channel are barely discernible. One potential strategy for improving temporal resolution is to increase the frame rate by acquiring smaller volumes. Although this method could theoretically maintain acquisition time, it would also reduce the FOV, which is undesirable for comprehensive sample imaging. Alternatively, increasing the repetition rate of the individual sub-volumes while maintaining the FOV might enhance temporal resolution but would extend the total acquisition time. Addressing these limitations requires advanced scanning and processing strategies capable of capturing both low- and high-frequency dynamics while preserving volume coverage and acquisition speed. One method to optimize the frequency bin selection could involve automatic binning algorithms^34^.

A significant challenge remains the limited FOV of the dynamic volumes. Since changing scan lenses is not a viable solution because of the required high lateral resolution, repositioning the scan head is necessary to cover larger tissue areas. Therefore, the study uses a motorized three‑axis linear robot with micrometer-level precision, enabling tissue scanning over an area of 2.6 × 2.6 mm^2^, with the flexibility to scan larger regions when needed. Integrating a seven-axis robotic system with an online probe-to-surface control for automatic height adjustments provides even greater adaptability^35^. However, the amount of data generated during imaging is a significantly limiting factor. Each raw volume, which consists of repeating sub-volumes, results in a data size of 480 GB. This poses a challenge for storage, processing, and real-time analysis. To mitigate this issue, it is essential to investigate novel scanning and processing strategies that enable the acquisition of a wide frequency range while minimizing acquisition time and storage size. The data storage process is currently constrained by the speed limitations inherent to our preliminary imaging software version, requiring approximately 23 min to save each dataset. To address this, the next development step involves integrating dynamic data processing and visualization into our real-time LURO‑OCT software^36^. This upgraded version supports parallel streaming and direct disk storage, significantly reducing storage time and improving workflow efficiency.

Further improvements in imaging speed could be achieved by reducing sampling density. Currently, the sample is oversampled by a factor of five in each scanning direction. However, a twofold oversampling would be sufficient, allowing for a theoretical scanning time reduction by a factor of nine. As recently demonstrated by Heldt et al., six repeated frames provide adequate dynamic contrast without compromising image quality^34^. Reducing the number of scanner revisitations could further optimize scanning efficiency by an additional factor of eight, enhancing overall imaging performance. This improvement could enable MHz-dOCT measurements of 1.4 × 1.4 mm^2^ in less than three seconds, facilitating real-time monitoring of dynamic biological tissue processes and potentially enabling whole-organ imaging, as demonstrated by Ma et al.^10^. The extension of MHz-OCT technology with dynamic contrast, combined with robotically assisted scanning, holds significant promise for pre- and post-transplant kidney imaging within a clinically feasible timeframe and may further enhance the system’s capabilities for future in vivo diagnostic applications.

## Methods

### MHz-dOCT setup and optical specifications

The MHz-dOCT system used in this work is an all-fiber-based system equipped with a home‑built FDML laser source and operated at an effective A-scan rate of 3.2 MHz, as previously demonstrated in^37,38^. The center wavelength is set to 1310 nm, and with a spectral bandwidth of 110 nm, a theoretical axial resolution of 9.4 µm and an imaging range of 5 mm in air is achieved. The OCT interference fringes are detected by a 1.6 GHz balanced photodetector (PDB480C-AC, Thorlabs Inc., USA), and its signal is acquired at 4 GS/s (ATS9373, AlazarTech, Canada).

**Fig. 4:**
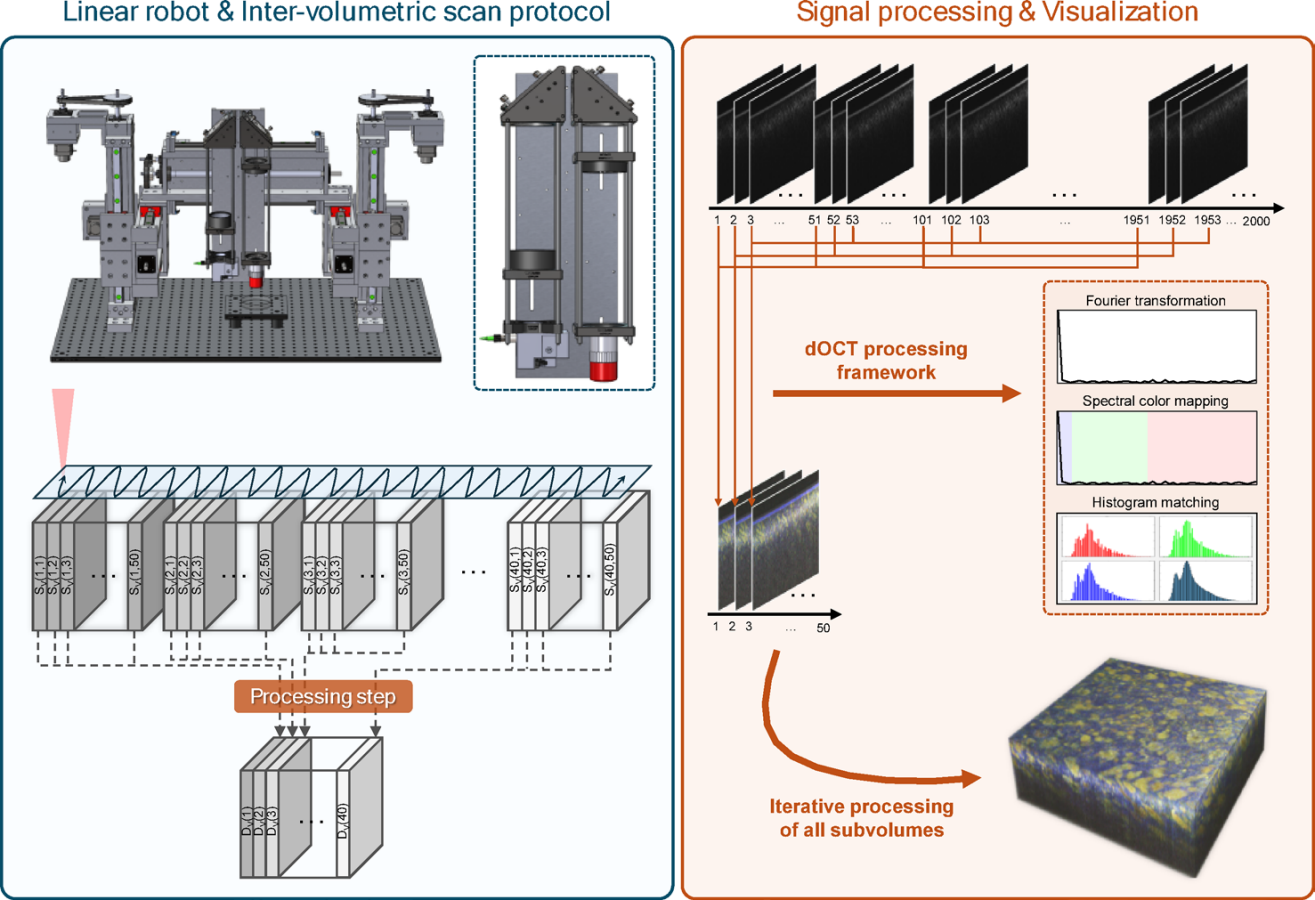
Schematic visualization of the volumetric dOCT principle and three-axis linear robot. The left blue box illustrates the design of the three-axis linear robot with the 4f optical system and the applied scan protocol to achieve inter-volumetric contrast using several subvolumes. The notation ${{\vec{S}}}_{{\vec{v}}}({\vec{i}},{\vec{n}})$ represents subvolumes, where ${\vec{i}}$ denotes the spatial index, and ${\vec{n}}$ indicates the temporal repetition number. ${{\vec{D}}}_{{\vec{v}}}({\vec{i}})$ refers to the dOCT volume. The right orange box displays the computation and visualization of the dynamic contrast. An exemplary three-dimensional dOCT volume of the porcine kidney is shown in the lower right corner.

The MHz-dOCT system contains a home-built scanning unit, which consists of a pair of galvanometric scanners (dynAXIS 421, Scanlab GmbH, Germany) and a fiber-collimator (F260APC-C, Thorlabs Inc., USA). Imaging was performed with two different objectives (M Plan Apo NIR 10X, M Plan Apo NIR 20X, Mitutoyo, Japan) to boost the lateral resolution compared to standard scan configurations of the OCT system with basic scan lenses. However, relay imaging of the scanning beam is required to fill the objective’s back aperture and fully utilize its resolving power. Therefore, two different 4f optical systems, comprising three spherical lenses (LA1399-C, LA1384-C, LA1256-C, Thorlabs Inc., USA), were set up between the scan unit and the objective to widen the initial scanning beam diameter according to the entrance pupil of the objectives. Two mirrors are used to fold the optical path and miniaturize the setup attached to a motorized three-axis linear robot. The scanning setup is visualized in Fig. 4. The measurements were performed on an actively isolated optical table to minimize external motion artifacts.

The resulting lateral resolution was characterized using a high-resolution test chart (USAF-1951 negative target #55-622, Edmund Optics, USA). The target’s second element of group 8, with a line pair width of 3.48 µm, was fully resolved with the 10 × objective setup. For the 20 × objective setup, we measured a lateral resolution of 2.76 µm. Detailed specifications of the two optical scan setups are listed in Table 1.

**Table 1:** Specifications of the different scan objectives.

Parameter	10x	20x
NA_Obj_	0.26	0.4
f_1_	87.5 mm	125 mm
f_2_	300 mm	300 mm
EP	9.6 mm	6.7 mm
z_R_	7.25 µm	3.72 µm
P_spl_	11.0 mW	12.6 mW
Res_lat_	3.48 µm	2.76 µm
FOV	1410 × 1460 µm^2^	1040 × 1060 µm^2^

NA_Obj_: Objective’s numerical aperture. f_1_: Focal distance lens (pair) 1. f_2_: Focal distance lens 2. EP: Entrance pupil beam diameter. z_R_: Calculated Rayleigh length. P_spl_: Power on sample. Res_lat_: Lateral resolution. FOV: Field of view.

### Design of the motorized three-axis linear robot for extended-area tissue scans

To enable extended-area tissue scanning, the scan unit with the 4f optical system is attached to a home-built, motorized, three-axis linear robot, facilitating extended-area mosaicking. Figure 4 illustrates its design. Using a combination of three individually controllable linear axes, the robot allows for precise and systematic scanning of extensive sample areas, covering approximately 20 × 20 × 20 cm. Five stepper motors (QSH4218-51-10-049-10 K, Analog Devices Inc., USA) and two triple-axis stepper motor driver boards (TMCM-3110-TMCL, Analog Devices Inc., USA) ensure accurate movement and alignment. Optical incremental encoders with a resolution of 10.000 lines per revolution (equivalent to 40.000 counts per revolution) provide precise motion control. A custom control software developed in LabVIEW (National Instruments, USA) synchronizes robot motion with the OCT imaging software for fully automated extended‑area scans.

The X-axis is supported by two Y- and Z-axes, providing increased stiffness and stability during the measurements. All five axes consist of a hollow CNC-milled frame with a C-shaped cross-section. Each is equipped with ball screw spindles (KGS-R-1605-RH-T5, NEFF Gewindetriebe GmbH, Germany) and preloaded ball nuts (KGF-D-1605-RH-EE, NEFF Gewindetriebe GmbH, Germany) to achieve minimum backlash. The spindles have a diameter of 16 mm and a pitch of 5 mm with a maximum deviation of 23 µm over a length of 300 mm. Profile rail guides with carriages are mounted along the hollow frames, with stopper blocks and limit switches (01050.5202-00, Marquardt GmbH, Germany) installed to prevent derailment. The Z-axes use 390 mm profile rail guides (EGR25R, HIWIN GmbH, Germany) with two carriages per rail (QEH25CAZ0H, HIWIN GmbH, Germany). For the X- and Y-axes, 340 mm rail guides (EGR25R, HIWIN GmbH, Germany) with preloaded carriages (QEH25CAZAH, HIWIN GmbH, Germany) are used.

The positioning accuracy was evaluated for various step sizes. Across all measurements, the mean error, including its standard deviation, did not exceed 1.46 µm ± 0.84 µm, demonstrating the system’s high precision and reliability.

### Imaging protocol and volumetric dynamic contrast calculation

In this study, we implemented an inter-volume scan protocol to enhance imaging efficiency while minimizing acquisition time. The key parameters of this scan protocol are listed in Table 2. Each volume was divided into 40 subvolumes comprising 2048 × 50 × 600 A-scans (XYZ‑dimensions). Unidirectional scanning was employed, with each subvolume acquired 50 times to ensure adequate temporal resolution. The chosen scan protocol was carefully optimized based on prior experimental evaluation of dynamic contrast quality, balancing acquisition time and frequency resolution. Parameters such as the number of B-scan repetitions and inter-scan intervals were systematically tested in advance to ensure sufficient contrast without compromising imaging throughput. Figure 4 outlines the scanning and processing workflow, where each acquired dOCT volume ${D}_{v}(i)$ is subdivided into 40 subvolumes ${S}_{v}(i,n)$. Here, $i$ indicates the spatial index of the subvolume, spanning from 1 to 40, while $n$ denotes the temporal repetition number, ranging from 1 to 50. The system operated at a frame rate of 612 Hz, resulting in a subvolume acquisition rate of 12.2 Hz and an approximate total scan time of 3.3 s. For extended FOV imaging, several volumes were acquired in an overlapping mosaic pattern using the three-axis linear robot.

**Table 2:** Key parameters of the scan protocol for one subvolume.

Imaging parameters	
Size	2048 × 50 × 600
Number	40
Repetition	50
Duration	82 ms
Frame rate	612 Hz
Volume rate	12.2 Hz

After acquiring all OCT datasets, the linear intensity images were processed using our research group’s custom LABVIEW (National Instruments Corp., USA) software. Due to scanner limitations, a high number of A-scans were acquired per B-scan. As the system operates at an A-scan rate of 3.2 MHz, this resulted in a temporal oversampling of approximately five times. Therefore, the A-scans were averaged twice, not to reduce noise but to simplify data handling and minimize data volume, halving the total number of A-scans. Subsequently, the datasets were resized and cropped along the Z-axis to retain only the relevant depth range. This reduced image noise and optimized the data for subsequent dOCT processing steps. Due to the twofold averaging, the volume was also resized along the Y-axis to maintain consistent spatial dimensions across all axes. Standard intensity images were exported on a logarithmic scale, and the same dataset in absolute intensity values for subsequent dOCT processing. For the standard intensity images, ten colocalized B-scans were averaged.

As displayed in Fig. 4, the dOCT process involves several individual steps and is realized in MATLAB (MATLAB R2023a, The MathWorks, Inc., USA). First, all colocalized B-scans are extracted from the dataset and registered frame-wise using MATLAB’s built-in *imregcorr* function to avoid motion artifacts. Then, a Fourier transform is applied to each spatial index to analyze the 50 temporally repetitive frequency characteristics. The resulting spectral data are divided into three distinct frequency ranges. For each voxel, the amplitude within each range are summed to compute the dynamic signal in that band. These three frequency bands are then used to color-code signal variations in the RGB color space. The blue color channel represents slow signal variations in the frequency range of 0 to 0.5 Hz, the green channel denotes medium frequency variations from 0.5 to 3 Hz, and the red channel indicates fast variations between 3 and 6 Hz. Each color channel is subsequently normalized and logarithmically transformed. An adaptive histogram equalization is then conducted using the standard deviation of the variations in the temporal OCT signal. The standard deviation is logarithmically transformed and used as the target histogram map. During the registration process, the dOCT volumes ${D}_{v}$ may undergo positional shifts along the z-axis, resulting in misalignment between ${D}_{v}$. Therefore, all volumes must be aligned and registered based on the reference volume ${D}_{v}(1)$ to ensure consistent height alignment across the entire dataset. After registration and dynamic contrast calculation of all frames, the combined dOCT subvolumes ${D}_{v}$ form a comprehensive three-dimensional dataset, covering an area of approximately 1.4 × 1.4 mm^2^ for the 10 × objective and 1 × 1 mm^2^ for the 20 × objective.

The volumes acquired with the three-axis linear robot are merged into an extended-area composite volume through a 3D registration and blending process. The histograms of the four volumes are first aligned using a reference column extracted from the *en face* image within the focal region of the first volume. Then, each volume is processed column by column, with histogram matching applied across all color channels. This approach ensures a smooth and consistent color distribution across the volumes, effectively compensating for any variations in image characteristics. The volumes are then registered using the open-source software *Elastix*^39^, which is built on the Insight Toolkit framework^40^. The software performed iterative optimization to minimize a cost function based on mutual information to quantify the similarity between the volumes and determine their alignment. Each volume is subsequently mapped onto a common coordinate system using its respective transformation. The volumes are then overlaid and blended using arithmetic averaging to minimize intensity differences in the overlapping regions. Next, hard edges are detected and smoothed to reduce artifacts and ensure smooth transitions. Any color casts caused by the histogram adjustments of the RGB volumes are corrected. Furthermore, intensity attenuation is applied along the z-direction to reduce signal artifacts in noise-dominated regions caused by the histogram-matching process.

### Ex vivo kidney tissue and histological preparation

Studies were performed on ex vivo porcine kidney tissues to assess the dynamic imaging performance of MHz-OCT. Freshly excised porcine kidneys, designated as slaughter by-products, were sourced from a local butcher. Immediately after excision, the samples were stored and transported in an ice-filled cooler to preserve tissue integrity, and imaging was performed within the first few hours post-mortem. For imaging, the tissue samples were cut into 12 mm segments using a biopsy puncher and put in biopsy embedding cassettes (Sanowa Laborprodukte GmbH, Germany). Following experimental procedures, samples were fixed in 4% buffered paraformaldehyde at 6 °C. Fixed tissues were then embedded in paraffin, sectioned into 5 µm slices using a microtome, stained with hematoxylin and eosin, and digitized using an automatic slide scanner (PANNORAMIC MIDI II, 3DHISTECH Ltd., Hungary).

**Fig. 5:**
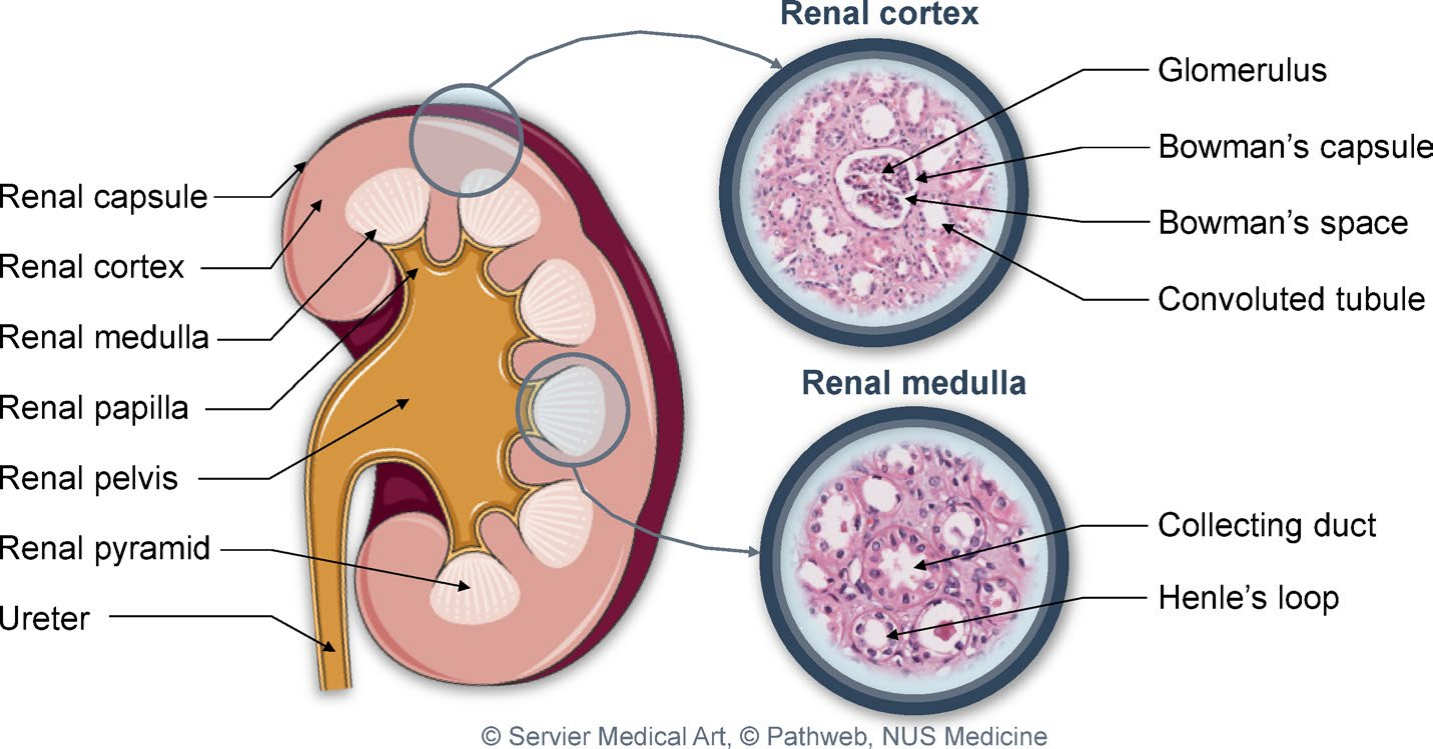
Localization of the examined kidney samples and relevant normal anatomical structures. The image was created using graphics from Servier Medical Art^41^ and high-quality histology slices with permission from Pathweb, NUS Medicine^42^.

In the imaging experiments, two kidney regions were examined, as illustrated in Fig. 5. The tissue sample from the renal cortex was excised from the surface and oriented upwards to mimic in vivo imaging conditions. For imaging of the renal medulla, a tissue sample was resected from the inner kidney at the location indicated in the figure. Figure 5 provides a schematic representation of kidney anatomy, including normal histological sections of the renal cortex and medulla, to support the detailed descriptions presented in the results section.

## Supplementary Information

Below is the link to the electronic supplementary material.

Supplementary Material 1

Supplementary Material 2

Supplementary Material 3

Supplementary Material 4

Supplementary Material 5

## Data Availability

The original OCT data used to generate the results reported in this paper are not publicly available due to their size, which amounts to several terabytes. They can be obtained from Prof. Robert Huber upon reasonable request.

## Abbreviations

dOCT: Dynamic optical coherence tomography
FDML: Fourier domain mode locking
FOV: Field of view
OCT: Optical coherence tomography

## Code availability

The code used to analyze the data and generate the plots for this paper is available from the corresponding author upon reasonable request.
