# Supplementary material for: Label-free volumetric imaging of porcine kidney tissue over extended areas using dynamic MHz-OCT
Source: Sci Rep. 2025 Sep 12;15:32426. doi: 10.1038/s41598-025-15032-6 (PMC12432236; doi:10.1038/s41598-025-15032-6)
Supplement: Supplementary file 1 — Supplementary Material 1 [file 41598_2025_15032_MOESM1_ESM.docx]

**Vid. S1 Stitched extended field of view dOCT of renal medulla.** The 3D-Volume consists of four sequentially scanned volumes with a lateral field of view of 1.4 x 1.4 mm² and approximately 15 % overlap. Imaging was performed using a 10x objective. Detailed descriptions of individual time points of the video:

0:00-0:20 – Slicing through successive *en face* cross-sections of all four dOCT volumes

0:21-0:25 – Schematic animation of the stitching process

0:26-0:37 – Slicing through successive *en face* cross-sections of the stitched dOCT volume

0:38-0:49 – Display of the stitched dOCT volume in 3D with 360° rotational view

**Vid. S2 Standard intensity and dynamic OCT of renal medulla using a 10x objective.** Detailed descriptions of individual time points of the video:

0:00-0:15 – Slicing through successive *en face* cross-sections of the OCT volume (left: dOCT, right: intensity OCT)

0:16-0:20 – Overlay of dynamic contrast and standard intensity of a single OCT *en face* cross-section with changing display of the color channels

0:21-0:26 – Display of the volume with overlayed color channels in 3D and with 360° rotational view

0:27-0:35 – Display of the volume in 3D and with 360° rotational view – side by side (left: dOCT, right: intensity OCT)

0:36-0:45 – Slicing through successive B-scans of the OCT volume (left: dOCT, right: intensity OCT)

**Vid. S3 Standard intensity and dynamic OCT of renal medulla using a 20x objective.** Virtual slicing through successive *en face* cross-sections of the OCT volume (left: dOCT, right: intensity OCT).

**Vid. S4 Dynamic OCT of the outer renal cortex.** Imaging was performed using a 20x objective. Detailed descriptions of individual time points of the video:

0:00-0:06 – Display of the dOCT volume in 3D with 360° rotational view

0:07-0:14 – Slicing through successive *en face* cross-sections of the dOCT volume

0:15-0:22 – Slicing through successive B-scans of the dOCT volume

0:23-0:30 – Slicing through successive YZ-cross-sections of the dOCT volume

0:31-0:47 – Zoom into a specific region showing detailed three-dimensional structures of renal corpuscles
